# Amyloid β peptide-induced inhibition of endothelial nitric oxide production involves oxidative stress-mediated constitutive eNOS/HSP90 interaction and disruption of agonist-mediated Akt activation

**DOI:** 10.1186/s12974-015-0304-x

**Published:** 2015-05-03

**Authors:** Folami Lamoke, Valeria Mazzone, Tiziana Persichini, Annamaria Maraschi, Michael Brennan Harris, Richard C Venema, Marco Colasanti, Micaela Gliozzi, Carolina Muscoli, Manuela Bartoli, Vincenzo Mollace

**Affiliations:** Department of Ophthalmology, Georgia Regents University, Health Sciences Campus, 1120 15th St., Augusta, GA 30912 USA; Department of Biology, University of Rome ‘Roma Tre’, Via Ostiense, 169, Rome, 00154 Italy; Department of Neurology and Laboratory of Neuroscience, IRCCS Istituto Auxologico Italiano, Cusano Milanino 20095, Milan, Italy; Department of Kinesiology, College of William and Mary, 200 Stadium Dr., Williamsburg, VA 23186 USA; Vascular Biology Center, Georgia Regents University, 1120 15th St., Augusta, GA 30912 USA; IRC-FSH, Department of Health Sciences, University of Catanzaro ‘Magna Graecia’, Catanzaro Complesso ‘Ninì Barbieri’, Roccelletta di Borgia, 88021 Italy; IRCCS San Raffaele Pisana, Via di Val Cannuta, 247, 00166 Rome, Italy

## Abstract

**Background:**

Amyloid β (Aβ)-induced vascular dysfunction significantly contributes to the pathogenesis of Alzheimer’s disease (AD). Aβ is known to impair endothelial nitric oxide synthase (eNOS) activity, thus inhibiting endothelial nitric oxide production (NO).

**Method:**

In this study, we investigated Aβ-effects on heat shock protein 90 (HSP90) interaction with eNOS and Akt in cultured vascular endothelial cells and also explored the role of oxidative stress in this process.

**Results:**

Treatments of endothelial cells (EC) with Aβ promoted the constitutive association of HSP90 with eNOS but abrogated agonist (vascular endothelial growth factor (VEGF))-mediated HSP90 interaction with Akt. This effect resulted in blockade of agonist-mediated phosphorylation of Akt and eNOS at serine 1179. Furthermore, Aβ stimulated the production of reactive oxygen species in endothelial cells and concomitant treatments of the cells with the antioxidant N-acetyl-cysteine (NAC) prevented Aβ effects in promoting HSP90/eNOS interaction and rescued agonist-mediated Akt and eNOS phosphorylation.

**Conclusions:**

The obtained data support the hypothesis that oxidative damage caused by Aβ results in altered interaction of HSP90 with Akt and eNOS, therefore promoting vascular dysfunction. This mechanism, by contributing to Aβ-mediated blockade of nitric oxide production, may significantly contribute to the cognitive impairment seen in AD patients.

## Introduction

Alzheimer’s disease (AD) is a progressive neurodegenerative disorder [1] characterized by the accumulation of intracellular neurofibrillary tangles and extracellular senile plaques of which the major component is the amyloid β peptide (Aβ) [2,3]. Although the molecular mechanisms leading to neuronal damage in AD have not been completely understood, it is well established that increased production of Aβ, in soluble and/or aggregate form, is a key causative event for AD [3,4].

A growing body of evidence has indicated that the cerebral vasculature is an important target of Aβ and that vascular dysfunction significantly contributes to neuronal damage and dementia [5,6]. AD patients have reduced cerebral blood flow. This precedes dementia and may contribute to its progression. Recently, it has been shown that endothelin-1 is elevated in Alzheimer’s disease and upregulated by amyloid β [7]. Cardiovascular risk factors, especially hypertension, have been associated with higher risk of developing Alzheimer’s disease, partially through cerebral vasculature impairment and reduced nitric oxide production [8]. The brains of patients with AD exhibit elevated levels of ACE, Ang-II, and angiotensin II receptors (AR-II) [9].

In addition, decreased endothelium-derived nitric oxide (NO) bioavailability and vascular dysfunction have been demonstrated in AD [10-15].

NO production at the endothelial cell level involves the activity of the enzyme endothelial nitric oxide synthase (eNOS, NOS III), which is constitutively expressed and produces NO in a calcium-dependent manner [16]. In models of chronic brain hypoperfusion, *in vivo* administration of Aβ has been shown to increase the expression of eNOS and, paradoxically, to decrease endothelium-derived NO formation [8], thus, suggesting that Aβ could affect the activity of this enzyme. eNOS post-translational modification, including phosphorylation at specific amino acid residues, can profoundly affect its activity and, therefore, influence NO production [17]. Furthermore, eNOS association with a specific set of interacting proteins has been shown to critically regulate its enzymatic activity by exerting both stimulatory and/or inhibitory effects [18-21]. In particular, the chaperone molecule heat shock protein 90 (HSP90) has been demonstrated to possess a key stimulatory role by maintaining the enzyme in an active conformational state and by facilitating its phosphorylation at serine 1177/1179 [22-24]. Aβ has been shown to inhibit eNOS phosphorylation at serine 1177/1179 and at other residues [25,26], however, no information is available about the effects of Aβ on eNOS interaction with HSP90 or other regulatory partners, which could potentially contribute to these inhibitory effects. In addition, increased production of reactive oxygen species (ROS) and consequent oxidative stress have been shown to negatively influence eNOS activity and significantly contribute to vascular dysfunction in a number of cardiovascular diseases including diabetes and hypertension [23-30]. Aβ-induced oxidative stress has been extensively documented [31-33]; however, its direct contribution to the reported effects in inhibiting eNOS-dependent NO production or in influencing its interaction with regulatory proteins is not clear.

In this study, we show that in bovine aortic endothelial cells soluble Aβ_1–42_ promotes the constitutive association of HSP90 with eNOS. This effect resulted in blockade of agonist-mediated phosphorylation of Akt and eNOS at serine 1179. These effects are correlated with Aβ’s ability to increase the production of hydroxyl radicals in endothelial cells and are reverted by concomitant treatment with the antioxidant N-acetyl-cysteine.

## Materials and methods

### Materials

All tissue culture reagents were from Invitrogen (Carlsbad, CA, USA), unless otherwise specified. Fetal bovine serum (FBS) was from Gemini Bio-products (Woodland, CA, USA). Aβ_25–35_, Aβ_35–25_, Aβ_1–42_, and Aβ_42–1_ peptides, as well as nitro-L-arginine methyl ester (L-NAME), were from Sigma-Aldrich (St. Louis, MO, USA). Monoclonal and polyclonal anti-eNOS and anti-HSP90 antibodies were from BD-Transduction Laboratories (San Diego, CA, USA). The polyclonal antibody for phospho-eNOS (Ser 1179) was from Invitrogen (Grand Island, NY, USA). Polyclonal and monoclonal antibodies for anti-Akt and phospho-Akt (Ser473) were purchased from Cell Signaling (Danvers, MA, USA). Protein A/G agarose beads were from Santa Cruz Biotechnology (Santa Cruz, CA, USA). The ECL chemiluminescence detection assay as well as the peroxidase-conjugated anti-mouse/anti-rabbit IgG were from Amersham Biosciences (Piscataway, NJ, USA). The cGMP enzyme-immunoassay kit was from Cayman Chemical Co. (Ann Arbor, MI, USA). The blots were reprobed after stripping using a stripping solution from Pierce (Rockford, IL, USA).

### Cell cultures and treatments conditions

Primary cultures of bovine aortic endothelial cells (BAEC) and brain endothelial cells (BBEC) were purchased from VEC Technologies (Rensselaer, NY, USA) or the endothelial cell Core Facility of the Vascular Biology Center at Georgia Regents University. BAEC were cultured in medium M199 in the presence of 10% FBS, 1% glutamine, 1% penicillin/streptomycin, and 1% non-essential amino acid and vitamin mixtures. The cells were used between passages 3 and 5. Oligomeric preparations of Aβ peptides (25–35, 35–25, 1–42, or 42–1) were prepared by re-suspension in serum-free medium, left overnight at room temperature, then sonicated before supplementation to the culture medium as described previously [34].

The cells were pretreated for 24 h with 1 μM Aβ_25–35_, 1 μM Aβ_35–25_, 5 μM Aβ_1–42_, and 5 μM Aβ_42–1_. These doses of Aβ and the time of exposure were chosen based on preliminary experiments testing maximal effects (data not shown). After pre-incubation the cells were stimulated with 20 ng/ml vascular endothelial growth factor (VEGF) for established time points. Rat aortic smooth muscle cells (RASMC) were also obtained from VEC Technologies and used between passages 2 to 5 for the cGMP experiments. The RASMC cultures were maintained in DMEM (Invitrogen, Grand Island, NY, USA) containing 10% FBS and 1% penicillin/streptomycin.

### Nitric oxide production

Nitric oxide release in BAEC was measured by cGMP reporter cell assay following a protocol modified from Ishii *et al*. [35,36]. Confluent BAEC and RASMC were serum-starved for 16 to 18 h and then incubated in Locke’s buffer (154 mM NaCl, 5.6 mM KCl, 2 mM CaCl2, 1 mM MgCl2, and 10 mM Hepes, pH7.4) for at least 20 min before the experiment. The BAECs were then stimulated with 20 ng/ml VEGF for 30 min and, after stimulation, the bathing medium was rapidly transferred to the confluent RASMC, let incubate for 3 min, then the cells were lysed in ice-cold 20-mM sodium acetate, pH 4.0. The cGMP concentration in RASMC treated with the BAEC conditioned medium was determined using an enzyme immunoassay kit following the manufacturer’s instructions.

### Determination of reactive oxygen species formation

The production of reactive oxygen species in endothelial cells, following the different treatments, was determined by a fluorimetric assay using 2,7-dihydrochlorofluorescein (DHCF) from Invitrogen (Grand Island, NY, USA). BAEC, and BBEC plated in 96-well plates, were cultured for 24 h in serum-free condition and in presence or absence of Aβ. After this time, the cells were incubated with 5 μM DHCF for 1 h. The hydroxyl radicals produced in response to the different treatments oxidize the DHCF and generate its oxidation fluorescent product dichlorofluorescein (DCF). The fluorescence intensity was measured with a multi-detection microplate reader (BioTek, Winooski, VT, USA) using an excitation and emission light at 485 nm and 535 nM, respectively. Some samples were also incubated in the presence of 50 U of cell permeable superoxide dismutase (PEG-SOD from Sigma-Aldrich, Saint Louis, MO, USA), which was used as negative control to assess the specificity of the assay.

### Immunoprecipitation and Western blotting

Western blotting analysis was performed as previously described [37,38]. Briefly, BAEC and BBEC were lysed with a modified RIPA buffer (20 mM Tris–HCl (pH 7.4), 2.5 mM EDTA, 50 mM NaF, 10 mM Na_4_P_2_O_7_, 1% Triton X-100, 0.1% sodium dodecyl sulphate, 1% sodium deoxycholate,1 mM PMSF, and 2 mM Na_3_VO_4_). The samples were then subjected to SDS-PAGE electrophoresis and immunoblotted on nitrocellulose (Schleicher & Schuell Biosciences Inc., Keene, NH, USA). Protein-protein interaction was determined by immunoprecipitation analysis performed as previously described [37,38]. For these experiments, the cells were lysed with a buffer containing: 25 mM Tris–HCl (pH 7.4), 2.5 mM EDTA, 50 mM NaF, 10 mM Na_4_P_2_O_7_, 1% Triton X-100, 1 mM PMSF, and 2 mM Na_3_VO_4_. The cell lysates were allowed to react overnight with the primary antibody and the immunocomplexes were then precipitated with pre-cleared protein A/G agarose beads. After washing three times with ice-cold washing buffer (0.1% Triton X-100 in TBS) the beads and the immunocomplexes were precipitated by centrifugation, solubilized by resuspension in 2X SDS-sample buffer and by boiling at 100°C for 5 min. All the densitometry units have been normalized against total enzyme for each lane and are expressed as the ratio of phosphorylated proteins to total.

## Results

### Aβ blocks eNOS phosphorylation at serine 1179 and eNOS-dependent NO production

**Figure 1:**
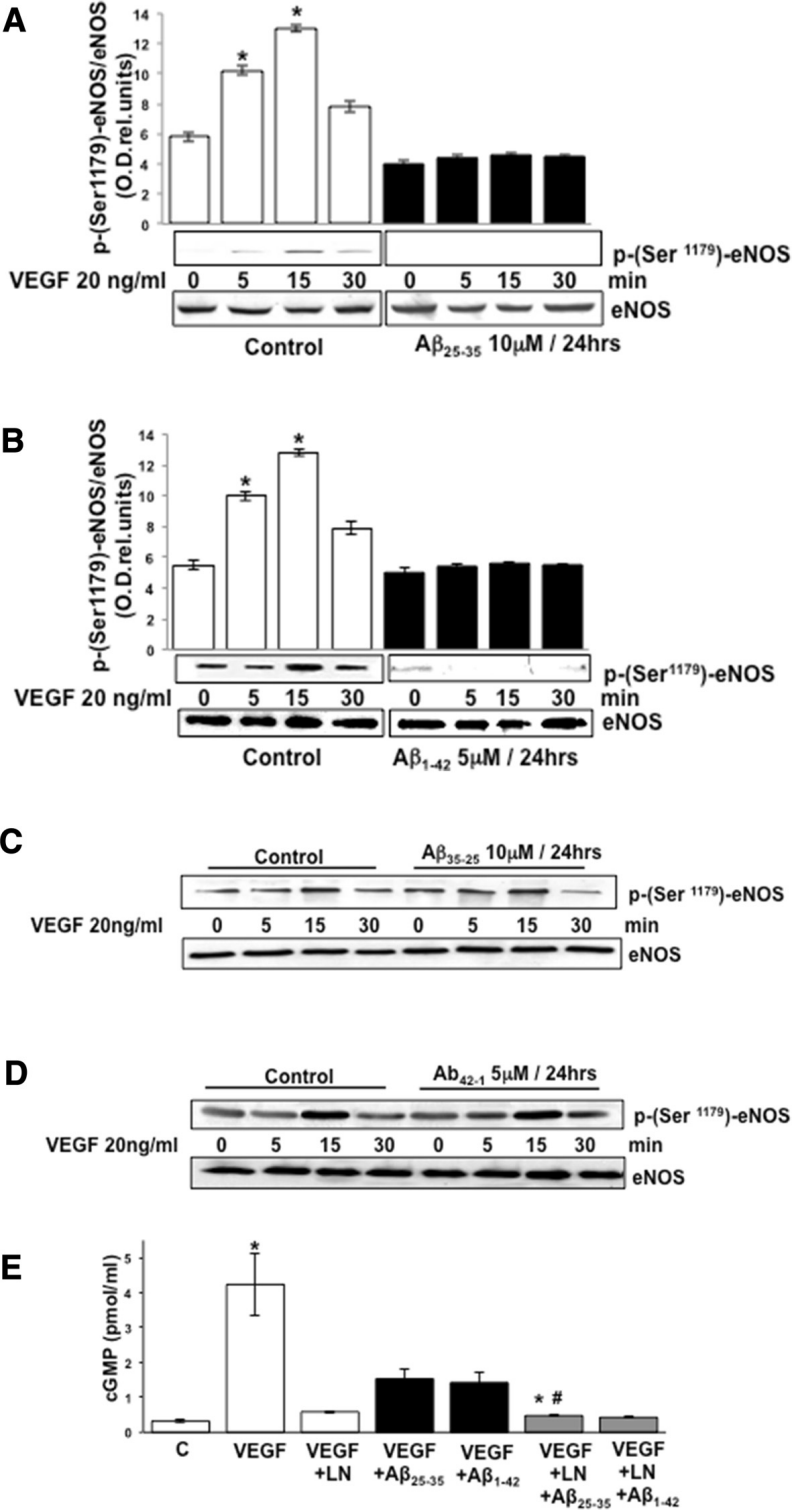
Western blotting analysis showing VEGF-stimulated (20 ng/ml for 0 to 30 min) eNOS phosphorylation at serine 1179 in BAECs cultured for 24 h in the presence (black bars) or absence (control, white bars) of 10 μM Aβ_25–35_ **(A)** and 5 μM Aβ_1–42_ **(B)**. Blots were subjected to densitometric analysis and the obtained data were analyzed for statistical significance (*P < 0.01 versus 0 min control group, *n* = 3). **(C-D)** Western blotting analysis demonstrating VEGF-stimulated eNOS phosphorylation at serine 1179 in BAECs cultured for 24 h in the presence of reversed inactive 10 μM Aβ_35–25_ **(C)** and 5 μM Aβ_42–1_ **(D)**. **(E)** Endothelial nitric oxide release. cGMP formation in BAECs cultured for 24 h in the presence (black bars) or absence (white bars) of 10 μM Aβ_25–35_ and 5 μM Aβ_1–42_ and following stimulation with 20 ng/ml VEGF for 30 min. cGMP formation, which directly reflects the nitric oxide release, was determined by reporter cell assay by measuring picomoles of cGMP generated in rat aortic smooth muscle cells after 3 min of exposure of the cells to media of BAECs after the different treatments, as indicated above and as explained in the text. To assess the assay specificity, some experiments were conducted with BAECs pre-stimulated with 100 μM of the nitric oxide synthase inhibitor L-NAME (LN). (*P < 0.05 versus control, *n* = 4, #*P* < 0.05 versus VEGF, *n* = 4). VEGF, vascular endothelial growth factor; eNOS, endothelial nitric oxide synthase; min, minutes.

We selected two amyloid β peptides to conduct our experiments: Aβ_25–35_, the shortest fibrillar fragment typically used in culture based on its capability of retaining the toxicity of the full length Aβ (1-40/42) peptides [39], and Aβ_1–42_, the disease-associated fragment found in patients with AD [40]. BAEC were cultured for 24 h in the presence of 10 μM Aβ_25–35_ or 5 μM Aβ_1–42_ and then challenged with 20 ng/ml VEGF for different time points (0, 5, 15, and 30 min). Figure 1A,B shows the Western blotting analysis determining phospho-serine 1179-eNOS formation in response to VEGF stimulation in BAEC cultured in the presence or absence (control) of Aβ_25–35_ (Figure 1A) or Aβ_1–42_ (Figure 1B). Densitometric analysis was used to quantify the obtained data and to assess statistical significance of the results (Figure 1A,B). VEGF induced a time-dependent increase in eNOS phosphorylation at serine 1179 in control cells, but this effect was completely blocked by the pre-treatment of the cells for 24 h with 10 μM Aβ_25–35_ or 5 μM Aβ_1–42_. To test for the specificity of inhibitory activities of Aβ_25–35_/Aβ_1–42_ on phosphorylation of eNOS, BAEC were treated with the reversed inactive peptides Aβ_35–25_ (Figure 1C) and Aβ_42–1_ (Figure 1D). As shown in Figure 1C,D, VEGF-induced eNOS phosphorylation at serine 1179 was unaffected by the treatment with Aβ_35–25_ or Aβ_42–1_, thus proving that eNOS inhibition was specifically elicited by the Aβ_25–35_ and Aβ_1–42_ sequence derived from the amyloid precursor protein. Next, to establish whether Aβ_25–35_/Aβ_1–42_ effects on serine 1179 phosphorylation resulted in impairment of eNOS activity, we evaluated cGMP formation as a measure of NO production by eNOS in a cell reporter assay. The obtained data are summarized in Figure 1E. The treatment with Aβ_25–35_ or Aβ_1–42_ reduced the amount of cGMP generated by RASMC incubated with the BAEC-conditioned medium by over 80% (*P* < 0.05, *n* = 4) (Figure 1E). This latter directly correlates with the amount of NO generated by the BAEC in response to VEGF stimulation. To control for the specificity of the cGMP assay, some BAEC cells were incubated in the presence of 100 μM of NOS inhibitor L-NAME before VEGF stimulation (Figure 1E). These data showed that the pretreatment of BAEC with L-NAME prevented an accumulation of cGMP in RASMC. The incubation of BAEC pre-treated with Aβ_25–35_ and Aβ_1–42_ with L-NAME resulted in further decrease in cGMP formation in RASMC (Figure 1E) suggesting that multiple signaling pathways contribute to eNOS activation by VEGF.

### Aβ stimulates constitutive eNOS/HSP90 complex formation

**Figure 2:**
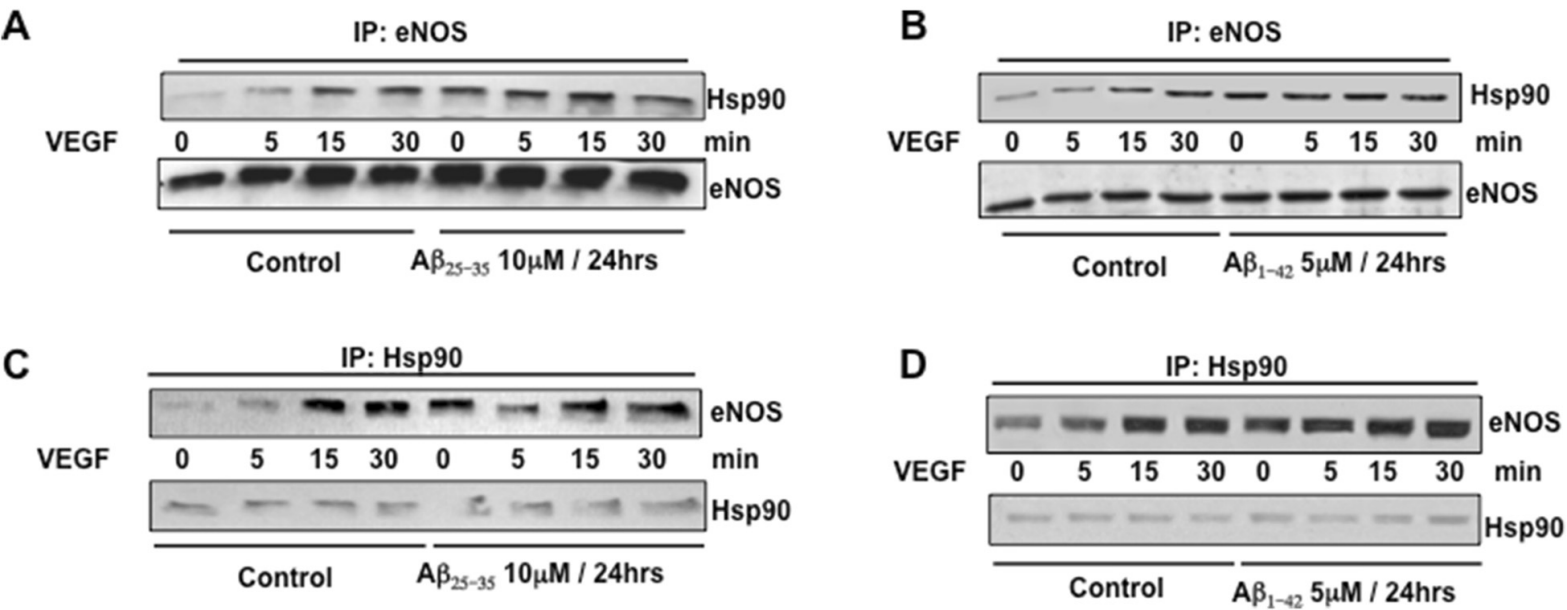
Immunoprecipitation studies showing time-dependent eNOS/HSP90 complex formation in response to stimulation with 20 ng/ml VEGF in BAECs cultured for 24 h in the presence or absence (control) of 10 μM Aβ_25–35_ **(A-C)** and 5 μM Aβ_1–42_ **(B-D)**. **(A-B)** Immunoprecipitation was carried-out with anti-eNOS antibody and the immunoprecipitated proteins were blotted with antibody against anti-HSP90. **(C-D)** Immunoprecipitation was carried-out with anti-HSP90 antibody and the immunoprecipitated proteins were blotted with antibody against anti-eNOS. VEGF, vascular endothelial growth factor; eNOS, endothelial nitric oxide synthase; min, minutes; Hsp90, heat shock protein 90.

eNOS activity is also influenced by its interaction with a specific set of regulatory proteins. Therefore, it is possible that Aβ peptides could alter the process of eNOS interaction with its regulatory partners. We tested this possibility and focused our attention on the chaperone molecule HSP90. In Figure 2, the results of the immunoprecipitation study assessing HSP90/eNOS complex formation are summarized. Specific immunoreactivity for HSP90 found in the protein complex immunoprecipitated by anti-eNOS antibody was increased in response to VEGF stimulation, thus indicating that VEGF stimulation of BAEC promoted the interaction of HSP90 with eNOS. This interaction, which peaked at 15 and 30 min after VEGF treatment (Figure 2A,B), was enhanced by the pre-treatment for 24 h with 10 μM Aβ _25–35_ and 5 μM Aβ _1–42_ and was also induced at the basal level as compared to untreated control cells (0 min on Figure 2A,B), thus suggesting that Aβ promotes the formation of eNOS/HSP90 complex in a constitutive manner. Consistently, specific immunoreactivity for eNOS was detected in response to VEGF treatment in a time-dependent manner in the protein complex immunoprecipitated by anti-HSP90 antibody (Figure 2C,D). The pre-treatment with either Aβ promoted eNOS/HSP90 interaction at the basal level (0 min) and further stimulated eNOS/HSP90 complex formation after stimulation with VEGF (Figure 2C,D).

### Aβ stimulates reactive oxygen species production

**Figure 3:**
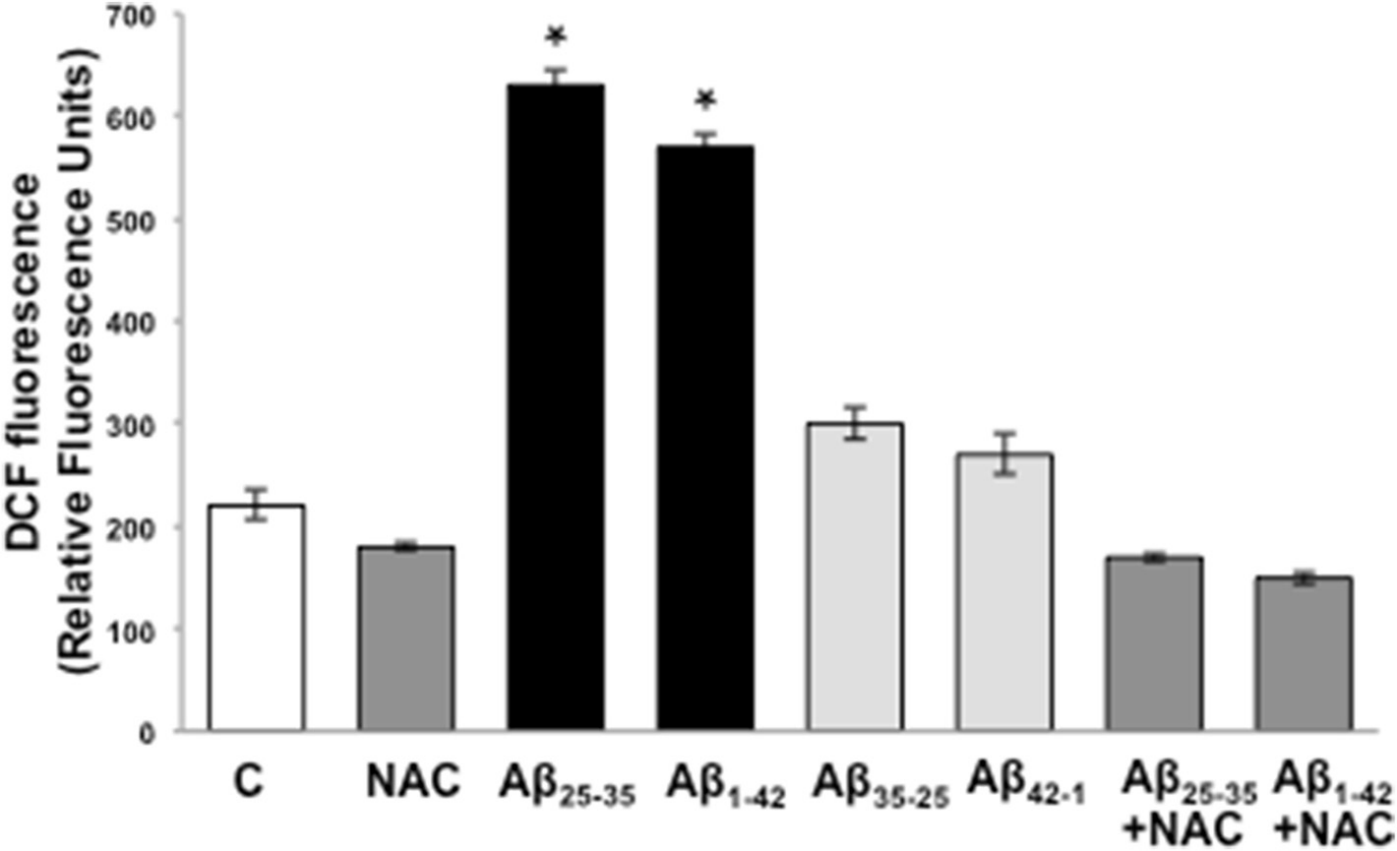
Effects of Aβ peptides (Aβ_25–35_/Aβ_1–42_ = active, Aβ_35–25_/Aβ_42–1_ = reverse, inactive) on superoxide anion production in BAECs assessed by dichloro-dihydrofluoresceindiacetate (DHCF-DA) fluorimetric assay. Some samples were pre-treated with 2 mM of the anti-oxidant N-acetyl-cysteine (NAC) to determine assay specificity. (*P < 0.001 versus control, *n* = 6).

Aβ has been shown to promote reactive oxygen species production and oxidative stress *in vivo* and *in vitro*. At the same time, oxidative damage has been involved in impairment of eNOS-dependent NO production and vascular dysfunction. To test the hypothesis that oxidative stress could be involved in Aβ effects on eNOS phosphorylation pattern or its interaction with HSP90, we first determined Aβ_25–35_/Aβ_1–42_ effects on production of superoxide anion in endothelial cells. Figure 3 illustrates the results obtained from a fluorimetric analysis measuring dichlorofluorescein (DCF) formation in BAEC cultured for 24 h in the presence of 10 μM Aβ_25–35_ or 5 μM Aβ_1–42_ in comparison to BAEC cultured in the presence of an equivalent dose of the reverse peptides Aβ_35–25_ and Aβ_42–1_ or control BAEC cultured in normal medium in the absence of Aβ. The conversion of 2,7-dihydrochlorofluorescein (DCHF) to the fluorescent compound dichlorofluorescein (DCF) is directly correlated to the amount of superoxide and hydroxyl radicals generated in the cells. Aβ_25–35_ and Aβ_1–42_ caused twofold increase (*P* < 0.001, *n* = 6) in ROS formation as compared with control BAEC, but this effect was not observed in cells cultured in the presence of the reversed inactive peptides Aβ_35–25_ or Aβ_1–42_ (Figure 3). In addition, Aβ_25–35_/Aβ_1–42_ induced ROS production was inhibited by the concomitant treatment of the cells with 2 mM of the antioxidant N-acetyl-cysteine (NAC), confirming the specificity of the fluorescent signal.

### Aβ effects on eNOS phosphorylation are blocked by N-acetyl cysteine

**Figure 4:**
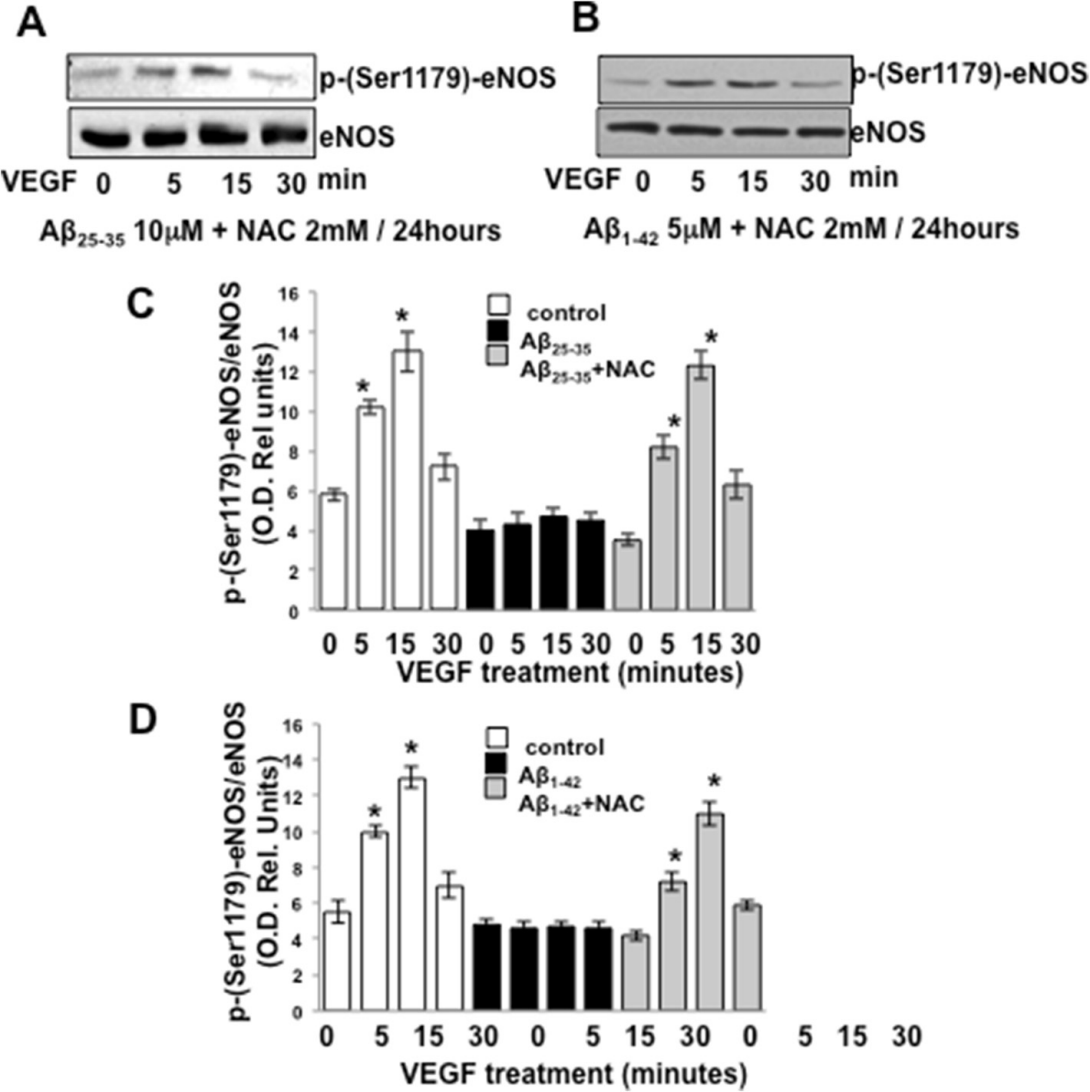
Representative Western blotting showing phosphorylation of eNOS at serine 1179 in response to stimulation with 20 ng/ml VEGF of BAECs cultured for 24 h in the presence of 10 μM Aβ_25–35_ **(A)** or 5 μM Aβ_1–42_ **(B)** in combination with 2 mM NAC. **(C-D)** The blots were subjected to densitometric analysis, and the obtained data were analyzed for statistical significance in comparison to the data shown in Figure 1A,B. (*P < 0.05 versus 0 min of each separate treatment group, *n* = 3). VEGF, vascular endothelial growth factor; eNOS, endothelial nitric oxide synthase; min, minutes; NAC, N-acetyl-cysteine.

Because of the observed ability of Aβ_25–35_/Aβ_1–42_ to augment the production of ROS in endothelial cells, we further tested whether antioxidants could prevent the effects of Aβ_25–35_ and Aβ_1–42_ in altering eNOS phosphorylation/activation pattern. BAEC were cultured for 24 h with 10 μM Aβ_25–35_ or 5 μM Aβ_1–42_ in the presence or absence of 2 mM NAC. Western blotting analysis was then conducted to determine the phosphorylation of eNOS at serine 1179 in response to stimulation with 20 ng/ml VEGF for 5, 15, and 30 min (Figure 4). As shown in Figure 4A,B, NAC treatment restored a normal pattern of eNOS phosphorylation at serine 1179 following VEGF stimulation, thus abolishing the inhibitory effect of Aβ_25–35_ and Aβ_1–42._ Densitometric analysis of the obtained results is plotted in Figure 4C,D where these data are compared to those shown in Figure 1A,B to assess statistical significance.

### NAC disrupts enhanced eNOS/HSP90 association by Aβ

**Figure 5:**
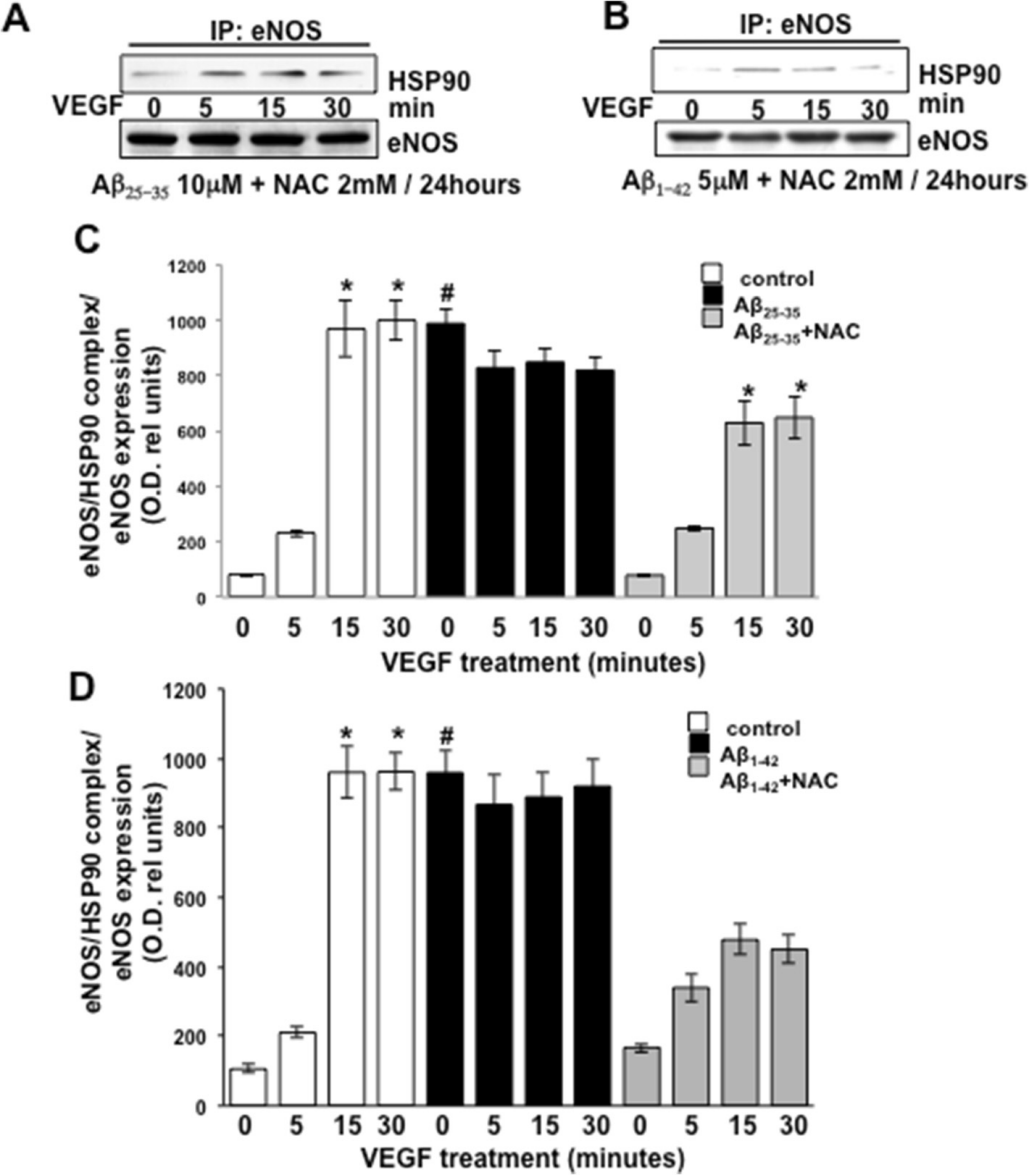
Immunoprecipitation analysis showing eNOS/HSP90 complex formation (as in Figure 2) in response to stimulation with 20 ng/ml VEGF of BAECs cultured in the presence of Aβ_25–35_ **(A)** and 1 μM Aβ_1–42_ **(B)** for 24 h and in combination with 2 mM NAC. **(C-D)** The blots were subjected to densitometric analysis, and the obtained data were analyzed for statistical significance in comparison to the data showed in Figure 2A,B. (*P < 0.05 versus 0 min of each separate treatment group and #*P* < 0.02 versus 0 min of untreated control cells (white bars), *n* = 4). VEGF, vascular endothelial growth factor; eNOS, endothelial nitric oxide synthase; min, minutes; NAC, N-acetyl-cysteine; Hsp90, heat shock protein 90.

Next then we determined the effects of the antioxidant NAC on Aβ_25–35_/Aβ_1–42_ induction of eNOS/HSP90 complex formation. As in the previous experiments (Figure 2) and as shown in Figure 5, treatment of the BAEC with NAC prevented the ability of Aβ_25–35_ (Figure 5A,C) and Aβ_1–42_ (Figure 5B,D) to promote HSP90/eNOS complex formation under basal conditions (0 min) and also restored an association pattern similar to that induced by VEGF in BAEC cultured under normal conditions without Aβ_25–35_ or Aβ_1–42_.

### Aβ inhibits VEGF-stimulated Akt phosphorylation at Ser473 and Akt/eNOS interaction

**Figure 6:**
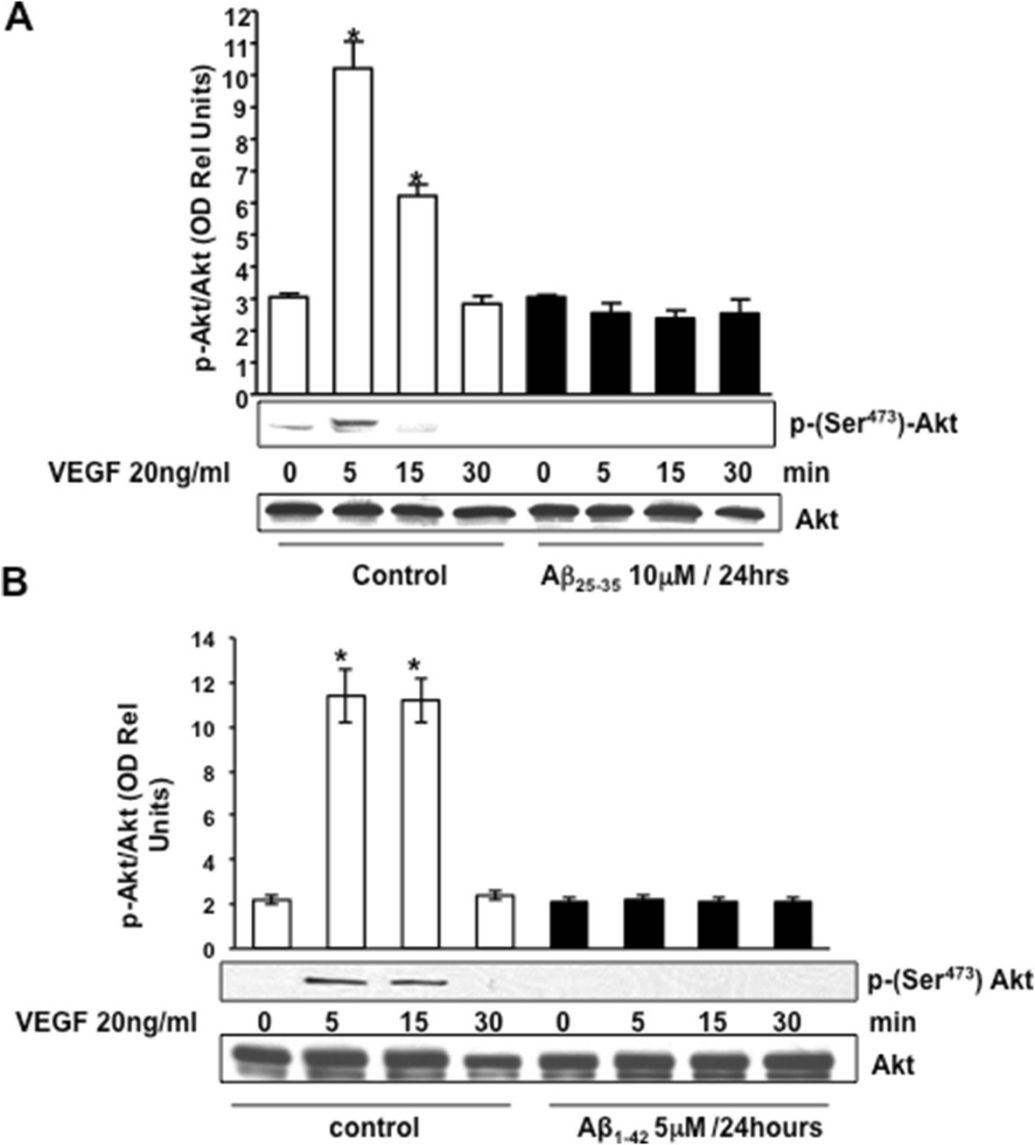
Representative Western blotting and densitometric analysis showing Akt phosphorylation at serine 473 in BAECs after stimulation with VEGF 20 ng/ml for 0 to 30 min. BAECs were cultured for 24 h in the presence (black bars) or absence (control, white bars) of 10 μM Aβ_25–35_ **(A)** and 5 μM Aβ_1–42_ **(B)**. Data were analyzed for statistical significance (*P < 0.01 versus 0 min control group, *n* = 3). VEGF, vascular endothelial growth factor.

**Figure 7:**
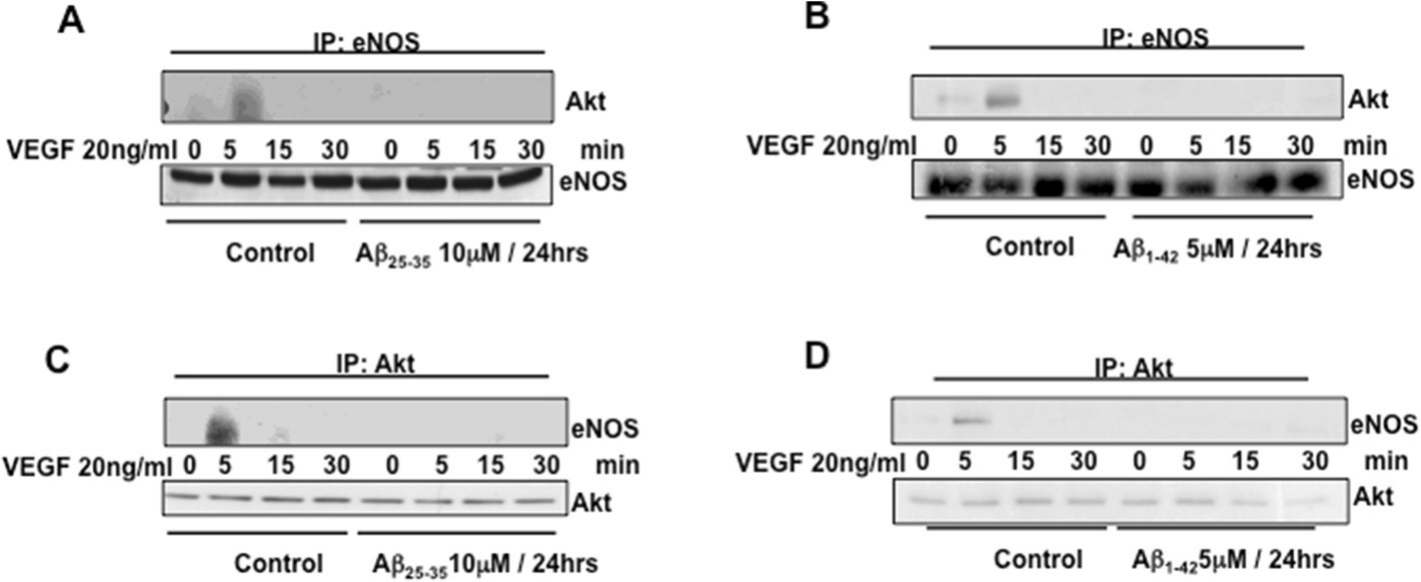
Immunoprecipitation studies showing time-dependent eNOS/Akt complex formation in response to stimulation with 20 ng/ml VEGF (0 min) in BAECs cultured for 24 h in the presence or absence (control) of 10 μM Aβ_25–35_ **(A-C)** and 5 μM Aβ_1–42_ **(B-D)**. **(A-B)** Immunoprecipitation was carried-out with anti-eNOS antibody, and the immunoprecipitated proteins were blotted with antibody against anti-Akt. **(C-D)** Immunoprecipitation was carried-out with anti-Akt antibody, and the immunoprecipitated proteins were blotted with antibody against anti-eNOS. VEGF, vascular endothelial growth factor; eNOS, endothelial nitric oxide synthase; min, minutes.

It is known that VEGF activation stimulates the protein kinase, Akt, and its subsequent phosphorylation/activation leads to the eNOS phosphorylation at Ser1179 [41] as detected in Figure 1. To determine whether Aβ affected the signaling cascade of Akt, we first investigated the effects of Aβ on Akt-Ser^473^ phosphorylation (Figure 6). As expected, VEGF significantly increased the phosphorylation of Akt-Ser473 in control cells. The phosphorylation of Akt peaked from 5 to 15 min and after 30 min was undetectable (Figure 6). The addition of Aβ_25–35_ (Figure 6) or Aβ_1–42_ (Figure 6B) resulted in the complete interruption in the sequential phosphorylation of Akt, which occurred in parallel to the disruption of eNOS activation observed previously (Figure 1A,B,E). Concurrently, we identified specific immunoreactivity for Akt in the protein complex immunoprecipitated by an anti-eNOS antibody which increased in response to VEGF stimulation (Figure 7A,B). This interaction peaked at 5 min after VEGF stimulation but was lost with the pre-treatment with Aβ_25–35_ (Figure 7A) and Aβ_1–42_ (Figure 7B) for 24 h. Consistent with those data, specific immunoreactivity for eNOS was detected in the protein complex immunoprecipitated by anti-Akt antibody after 5 min of VEGF stimulation (Figure 7C,D). The pre-treatment with either Aβ led to the loss of eNOS/Akt interaction. Obtained results validate that VEGF stimulation of BAEC promotes the interaction of Akt with eNOS and suggest that Aβ-induced blockade of eNOS activity and NO production involves the alteration in Akt/eNOS regulation.

### NAC recovers Aβ-induced Akt phosphorylation

**Figure 8:**
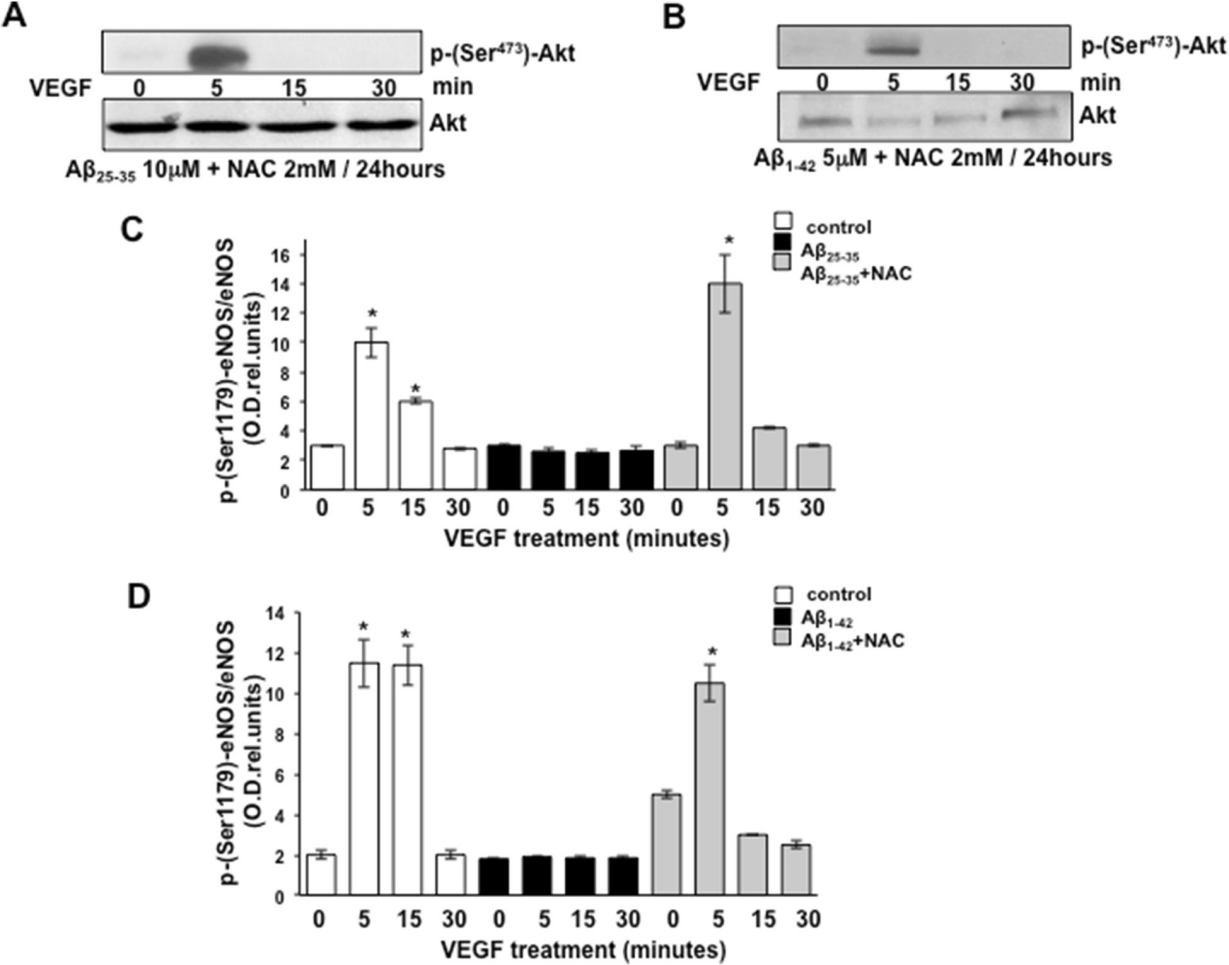
Representative Western blotting showing phosphorylation of Akt at serine 473 in response to stimulation with 20 ng/ml VEGF of BAECs cultured for 24 h in the presence of 10 μM Aβ_25–35_ **(A)** or 5 μM Aβ_1–42_ **(B)** in combination with 2 mM NAC. **(C-D)** The blots were subjected to densitometric analysis, and the obtained data were analyzed for statistical significance in comparison to the data shown in Figure 2A,B. (*P < 0.05 versus 0 min of each separate treatment group, *n* = 3). VEGF, vascular endothelial growth factor; eNOS, endothelial nitric oxide synthase; min, minutes; NAC, N-acetyl-cysteine.

We examined the effects of NAC on Akt phosphorylation at Ser473 by Western blotting when stimulated with 20 ng/ml VEGF for 5, 15, and 30 min. Treatment with the antioxidant NAC re-established the previously identified pattern of Akt phosphorylation following VEGF stimulation (Figure 8A,B). To assess statistical significance, the densitometric analysis data are compared to those shown in Figure 6A,B and the obtained results are plotted in Figure 8C,D.

### Aβ perturbs Akt/HSP90 association

**Figure 9:**
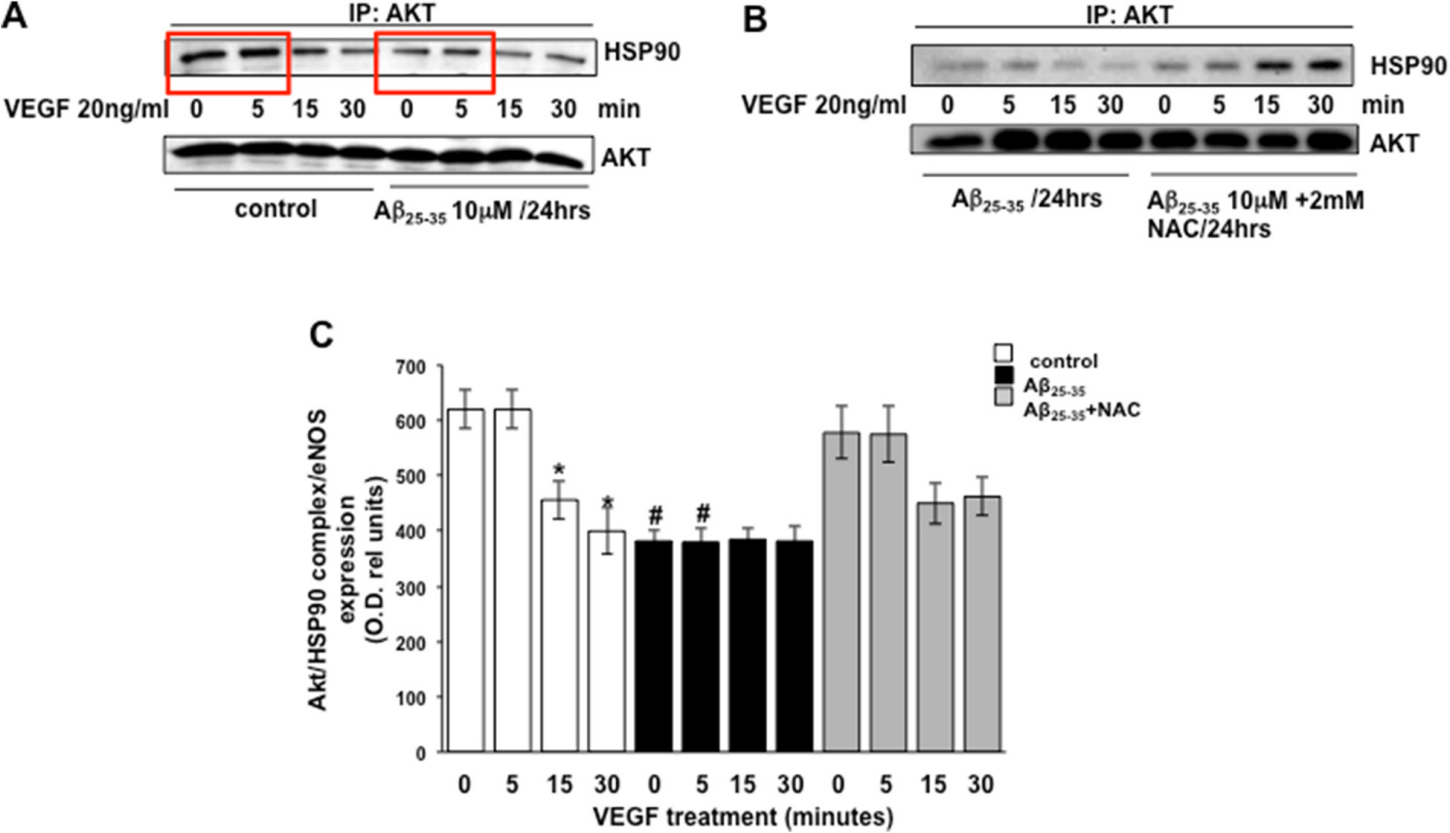
Akt/HSP90 complex formation detected by immunoprecipitation analysis in response to stimulation with 20 ng/ml VEGF of BAECs cultured in the presence of Aβ_25–35_ **(A)** for 24 h and in combination with 2 mM NAC **(B)**. Densitometric analysis of the blots analyzed for statistical significance are reported in the bar graphs **(C)** (*P < 0.05 versus 0 min of each separate treatment group and #*P* < 0.02 versus 0 min of untreated control cells (white bars), *n* =4). VEGF, vascular endothelial growth factor; eNOS, endothelial nitric oxide synthase; min, minutes; NAC, N-acetyl-cysteine; HSP90, heat shock protein 90.

**Figure 10:**
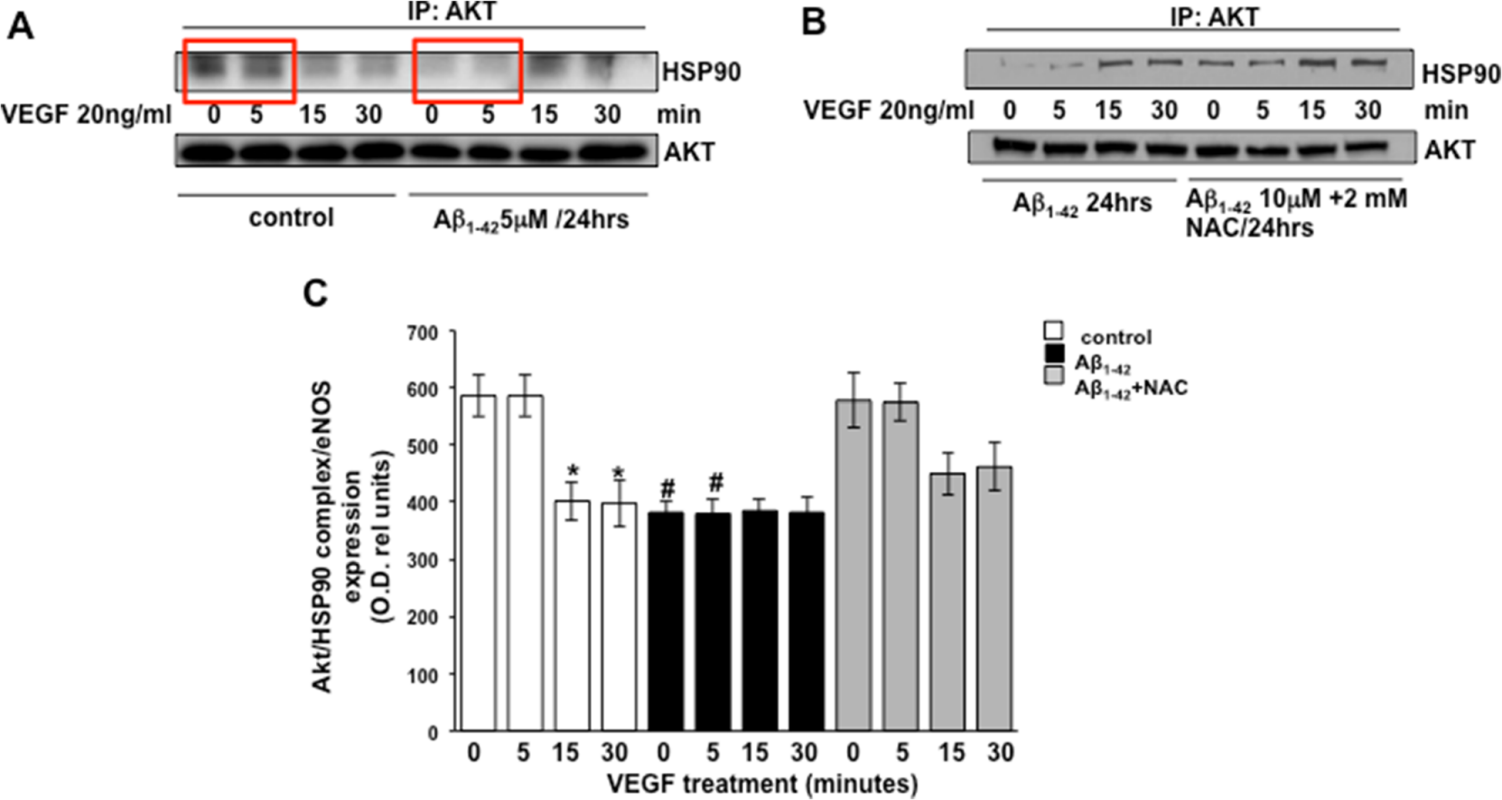
Akt/HSP90 complex formation detected by immunoprecipitation analysis in response to stimulation with 20 ng/ml VEGF of BAECs cultured in the presence of Aβ_1–42_ **(A)** for 24 h and in combination with 2 mM NAC **(B)**. Densitometric analysis of the blots analyzed for statistical significance are reported in the bar graphs **(C)** (*P < 0.05 versus 0 min of each separate treatment group and #*P* < 0.02 versus 0 min of untreated control cells (white bars), *n* = 4). VEGF, vascular endothelial growth factor; eNOS, endothelial nitric oxide synthase; min, minutes; NAC, N-acetyl-cysteine; HSP90, heat shock protein 90.

The replacement of HSP90 with its associated proteins can be critical to the regulation of those proteins, as demonstrated in studies that showing that nitrated lipid, OLA-NO_2_, enhances eNOS/Hsp90 interaction by replacing the eNOS/cav1 interaction, resulting in increasing eNOS activity [42]. We sought to determine whether an Aβ-induced augmentation of eNOS/HSP90 had an effect on Akt/HSP90. Our results show that both Aβ peptides block the basal ratio of VEGF-mediated Akt/HSP90 interaction at 5 min, the critical time point identified earlier as the window for Akt phosphorylation (Figure 9A,C and Figure 10A,C, red boxes). NAC effectively thwarts this abrogation, restoring Akt/HSP90 association to exceed a threshold where Akt activation can occur (Figure 9B,C and Figure 10B,C). To assess statistical significance, the densitometric analysis data are compared to those shown in Figures 9 and 10A,B and the obtained results are plotted in Figures 9 and 10C.

## Discussion

Endothelial production of nitric oxide is critical to the maintenance of vascular tone and the blood brain barrier, as well as to the anti-inflammatory and anti-thrombotic properties of the vascular endothelium [27]. Altered eNOS-dependent NO production results in endothelial dysfunction, which is associated with cardiovascular disease and has been shown to play a pathogenic role in AD [7,27].

In this study, we demonstrated that exogenous administration of the biologically active fragments Aβ_25–35_ and Aβ_1–42_ to cultured endothelial cells results in the production of reactive oxygen species (Figure 3), blockade of agonist-stimulated phosphorylation of eNOS at serine 1179 and decreased NO production (Figure 1). These effects correlated with Aβ-induced promotion a constitutive interaction of eNOS with its regulatory partner HSP90 (Figure 2). This correlated with a decrease in the interaction of HSP90 with Akt, the kinase responsible eNOS phosphorylation at Set1179 (Figure 9). These observed alterations appear to be the result of oxidative damage because the concomitant treatment of the cells with the antioxidant N-acetyl cysteine reverts Aβ effects restoring a normal pattern of eNOS phosphorylation at serine 1179 and interaction with HSP-90 (Figures 4 and 8). Our data demonstrated for the first time an inverse effect of Aβ on HSP90/eNOS interaction and HSP90/Akt interaction. We confirmed that upon stimulation with VEGF, Akt phosphorylates eNOS in an HSP90-dependent manner; synergistically increasing eNOS activity (Figures 1, 6, and 7). Once Aβ is introduced to these conditions, there is a diminished interaction between HSP90 and Akt, suggesting that the chaperone remains bound to eNOS in a manner that facilitates its inability to sufficiently bind Akt to signal its phosphorylation at Ser473 (Figures 9 and 10). Although the partial loss of this interaction could occur from other unexplored mechanisms, such as loss of the overall HSP90 protein level previously demonstrated as an effect of Aβ stimulation [43], it is reasonable to conclude that blocking the effects of Aβ can restore this interaction. In fact, when treated with NAC, HSP90/Akt interaction returns to basal levels and the detection of phospho-Akt (Ser^473^) indicate that this kinase overcomes the effects of Aβ in the presence of an antioxidant (Figures 9 and 10).

The results of our study strongly support the growing evidence that vascular dysfunction is involved in the etiology of AD dementia. Indeed, severe vascular changes, such as microvascular degeneration and breakdown of the blood brain barrier, have been demonstrated in AD patients [44]. In addition, Aβ can be found both in the brain parenchyma and in the cerebral vasculature of AD patients together with increased presence of monocytes/macrophages in the vessel wall and activated microglial cells in the parenchyma [45,46].

To date, attention has been focused on the molecular mechanisms by which Aβ and/or its fragments exert their effect on cells. The assumption that amyloid deposits are typically extracellular accounts for an effect at the level of the plasma membrane, possibly receptor-mediated. However, intracellular Aβ accumulation has been observed in various cell types, including neurons and endothelial cells [47-49]. In particular, it is not clear whether the effect of Aβ on eNOS activity initiates with the extracellular Aβ deposition or results from Aβ intracellular accumulation.

In this respect, reports have shown that internalized Aβ prevents eNOS from utilizing NADPH [49], a co-factor required for the activity of the enzyme. Furthermore, other studies have shown that over-expression of Aβ in endothelial cells or administration of exogenous (extracellular) Aβ on isolated vessels, blunts agonist-mediated eNOS activation and its phosphorylation at serine 1177/1179 [25,26]. Although these studies provide critical information, it is not clear if the aberrant intracellular production of Aβ peptides is a primary event in endothelial cells or if acute administration of extracellular Aβ represents the condition seen in AD patients that are chronically exposed to Aβ. It is tempting to speculate that both observations may be considered true and complementary. In particular, acute oxidative-independent effects of intra- or extracellular Aβ on eNOS activity may be transient, but could result in elevation of reactive oxygen species and in the oxidative-dependent chronic effects that we observe after 24 h of exposure.

Interestingly, our data show that Aβ promotes eNOS/HSP90 complex formation in a constitutive manner, providing a novel and important information. Previous work demonstrated that disruption of HSP90 interaction with eNOS results in inhibition of this enzymatic activity [22,50], therefore, one could expect that in the condition of vascular dysfunction the interaction of eNOS with its chaperone would be inhibited. However, this does not appear to be the case, as has been also shown in a model of diabetes [51]. Several reports have demonstrated enhanced HSP90 expression as part of the cellular response to oxidative stress conditions [52-54]. Specifically, the pathology of AD is characterized by enhanced heat shock proteins expression and activity in response to aberrant ‘misfolded’ proteins [55]. Of interest, HSP90 expression is induced by Aβ in glial cells which participate in the Aβ clearance process [56]. Our results demonstrate that Aβ might induce similar effects in endothelial cells. Moreover, HSP90 has been demonstrated to inhibit O^2−^ production by NOS isoforms [57,58], thus the enhancement of HSP90 interaction with eNOS, as shown by our results (Figure 2), could be a compensatory response to maintain the enzyme in a functional state in oxidative stress conditions when the risk of eNOS uncoupling is higher. However, this fails to occur, suggesting that other mechanisms are involved in this process and may be critical to the ability of HSP90 to regulate eNOS activity.

The evidence provided by our study extends and supports previous work demonstrating that Aβ promotes vascular dysfunction by impairing eNOS-dependent NO production. Furthermore, our study underscores the role of oxidative stress in the dysfunctional effects of Aβ on eNOS activity and function.
